# Effect of Chronic Valproic Acid Treatment on Hepatic Gene Expression Profile in *Wfs1* Knockout Mouse

**DOI:** 10.1155/2014/349525

**Published:** 2014-04-01

**Authors:** Marite Punapart, Mall Eltermaa, Julia Oflijan, Silva Sütt, Anne Must, Sulev Kõks, Leonard C. Schalkwyk, Catherine Fernandes, Eero Vasar, Ursel Soomets, Anton Terasmaa

**Affiliations:** ^1^Institute of Biomedicine and Translational Medicine, Department of Physiology, University of Tartu, 19 Ravila Street, 50411 Tartu, Estonia; ^2^Centre of Excellence for Translational Medicine, 19 Ravila Street, 50411 Tartu, Estonia; ^3^SGDP, Institute of Psychiatry at the Maudsley, King's College London, De Crespigny Park, London SE5 8AF, UK; ^4^Institute of Biomedicine and Translational Medicine, Department of Biochemistry, University of Tartu, 19 Ravila Street, 50411 Tartu, Estonia

## Abstract

Valproic acid (VPA) is a widely used anticonvulsant and mood-stabilizing drug whose use is often associated with drug-induced weight gain. Treatment with VPA has been shown to upregulate *Wfs1* expression *in vitro*. Aim of the present study was to compare the effect of chronic VPA treatment in wild type (WT) and *Wfs1* knockout (KO) mice on hepatic gene expression profile. Wild type, *Wfs1* heterozygous, and homozygous mice were treated with VPA for three months (300 mg/kg i.p. daily) and gene expression profiles in liver were evaluated using Affymetrix Mouse GeneChip 1.0 ST array. We identified 42 genes affected by *Wfs1* genotype, 10 genes regulated by VPA treatment, and 9 genes whose regulation by VPA was dependent on genotype. Among the genes that were regulated differentially by VPA depending on genotype was peroxisome proliferator-activated receptor delta (*Ppard*), whose expression was upregulated in response to VPA treatment in WT, but not in *Wfs1* KO mice. Thus, regulation of *Ppard* by VPA is dependent on *Wfs1* genotype.

## 1. Introduction

Valproic acid (VPA) is a widely used mood stabilizer and anticonvulsant [1]. In addition to VPA's effect of alleviating mania in the treatment of bipolar disorder (BD) there are several secondary metabolic side effects associated with VPA treatment, namely, a higher risk of developing insulin resistance and weight gain [2]. Weight gain has been reported nearly in half of the patients using VPA as a treatment [3]. In the case of BD patients, drug-induced weight gain is particularly noteworthy since overweight and several other metabolic disturbances are more common among people with BD compared to the general population [4]. The mechanism of VPA treatment-induced weight gain is unknown.

Impaired endoplasmic reticulum (ER) stress response was proposed to be associated with BD [5]. X box binding protein (XBP1) is a transcription factor of the ER stress response pathway. A mutation in this gene (−116C/G) is associated with bipolar disorder in the Japanese population [6] and* XBP1* expression is reduced in patients with BD [7, 8]. Wolframin* (WFS1) *is one of the genes that is induced in response to ER stress via XBP1 [9]. It has been shown that mood stabilizers lithium and VPA facilitate the ER stress response [10] and VPA induces* WFS1* expression* in vitro* [11]. VPA has also been shown to regulate the expression of other members of the ER stress pathway* in vitro* [12, 13] and* in vivo* [14].

Wfs1 is a 890 amino acid long transmembrane protein located in the ER. Lack of WFS1 function results in impaired ER stress response and apoptosis [9, 15–17]. Homozygous mutations in the* WFS1* gene result in a rare disease—Wolfram syndrome that is characterized by early-onset* diabetes mellitus*, progressive optic atrophy,* diabetes insipidus*, and deafness [18, 19]. The frequency of heterozygous carriers of mutations in the* WFS1* gene is remarkably high—1% of the general population [20] and heterozygosity for the* WFS1* mutations has been reported to be a significant risk factor for psychiatric illnesses [20, 21]. Mutations in the* WFS1* gene have been reported in patients with bipolar disorder, major depression, schizophrenia, and suicide victims without Wolfram syndrome [18, 19, 22–31]. There are conflicting reports on the connection between the* WFS1* gene and bipolar disorder. Kato et al. found no association of* WFS1* polymorphisms and expression level in postmortem tissue of Japanese BD patients [32], and similar results were found in another study in Japanese patients [27]. Nevertheless, a recent meta-analysis of genome-wide expression studies on BD revealed* WFS1* to be significantly correlated with BD in the prefrontal cortex [33].* Wfs1* KO mice were suggested as a possible animal model of BD [34]. We therefore hypothesize that the lack of Wfs1 function in* Wfs1* KO mice mimics to a certain extent the aberrant ER stress response observed in some patients with BD.

*Wfs1* KO mice exhibit impaired glucose tolerance and they are significantly smaller than their wild type littermates despite elevated growth hormone (GH) and insulin-like growth factor (IGF-1) levels [35]. In our previous study we found that acute treatment with valproic acid normalizes glucose tolerance in* Wfs1* mutant mice [36]. This effect of VPA was not mediated via increased insulin secretion, since the effect of VPA was also observed in mice with streptozotocin-induced type 1 diabetes. Thus, acute VPA treatment mimics and potentiates the effect of insulin in diabetic mice [36]. This study was conceived to investigate the effect of chronic administration of VPA on glucose tolerance and also on the gene expression in a metabolically relevant tissue. The liver was chosen as this organ plays an important role in the effect of insulin on the regulation of glucose metabolism, and also the expression level of* Wfs1* is substantial in the liver [37].

As male* Wfs1* KO mice exhibit stronger phenotype than female mice and* Wfs1* KO mice are smaller than wild type littermates the study was done on male young mice and a three-month long treatment was chosen to evaluate the possible effect of chronic VPA treatment on the growth of* Wfs1* mutant mice.

By comparing drug-induced changes of gene expression in wild type and* Wfs1* KO mice we hoped to find the genes that are potentially involved in the VPA treatment-induced metabolic alterations seen in BD patients.

## 2. Materials and Methods

### 2.1. Animals

Mice were housed under standard laboratory conditions on a 12 h light/dark cycle (lights on at 07:00 AM) with free access to regular chow diet (R70 Lantmännen, Sweden) and water. All animal experiments in this study were performed in accordance with the European Communities Directive (86/609/EEC) and a permit (number 39, October 7, 2005) from the Estonian National Board of Animal Experiments. Male wild type and* Wfs1* mutant mice were used throughout this study; they were 4 to 6 weeks old at the beginning of the experiment. Mice were treated for three months with valproic acid (VPA, Sigma Aldrich, 300 mg/kg i.p. daily) or vehicle (0.9% saline 10 mL/kg i.p. daily). Dose of VPA for chronic study was chosen as described previously [14]. A glucose tolerance test (2 g/kg i.p.) was performed 24 hours after the last VPA injection and mice were killed 24 hours after the glucose tolerance test. The liver was dissected out, snap frozen in liquid nitrogen, and stored at −80°C until further analysis. Mice were 16 to 18 weeks old when killed. Each experimental group consisted of 8 animals. Generation of* Wfs1* mutant mice was described previously [35].

### 2.2. Glucose Tolerance Test

Mice were kept in their home cages with free access to food and water. Food was removed 60 minutes prior to the experiment. Basal levels of blood glucose were determined from the tail vein; thereafter mice were injected with glucose (2 g/kg, i.p.) and blood glucose levels were determined using a hand held glucose meter (Accu-Check Go, Roche, Mannheim, Germany) at 30, 60, and 90 minutes following glucose injection.

### 2.3. Preparation of RNA and Microarray Hybridization

Total liver RNA was extracted using Trizol reagent (Ambion, Life Technologies). Integrity of total RNA was evaluated using the Agilent Bioanalyzer 2100 system (Agilent Technologies, CA, USA) and was within RNA integrity number (RIN) 7 to 9 and thus considered suitable for further processing. 300 nanograms of total RNA were processed to produce fragmented biotin-labeled cRNA using the Ambion WT expression kit according to manufacturer's instructions. Samples were hybridized Affymetrix GeneChip Mouse Gene 1.0 ST arrays and quantified. Images were processed and cell intensity files (CEL files) were generated in the GeneChip Command Console Software (Affymetrix). CEL files were processed using Expression Console v.1.1.2800.28061 to yield RMA summarized Log2 transformed expression values for probesets (CHP files). Normalized expression data (CHP files) were analysed using ANOVA in R (genotype x treatment) using R package Bioconductor. The data discussed in this publication have been deposited in NCBI's Gene Expression Omnibus [38] and are accessible through GEO Series accession number GSE55143 (http://www.ncbi.nlm.nih.gov/geo/query/acc.cgi?acc=GSE55143).

### 2.4. Microarray Data Analysis

Raw data from gene chips were processed with the RMA method, which involves quantile normalization. Two-way ANOVA was performed on the normalized expression data using R software. A gene list was created that contained probesets with *P* < 0.001 for genotype effects. Only genes showing significantly different changes greater than 2-fold were considered for effect of genotype. For the effect of VPA treatment a *P* value of 0.001 was used as a cutoff. Given the small number of genes for which genotype-treatment interaction was established, a *P* value of 0.003 was used as a cutoff.

Differently expressed genes were annotated to find the molecular function using the web-based international database Mouse Genome Informatics Gene Ontology (MGI GO) that includes genetic, biological, and genomic information of laboratory mouse and also the UniProt Knowledgebase (UniProtKB/Swiss-Prot) that holds the functional information of known proteins.

### 2.5. Gene Expression Studies with qRT-PCR Analysis

For confirming differences in expression of genes of interest found on gene chip, quantitative real-time PCR (qRT-PCR) analysis was used. For that purpose, the ABI PRISM 7900HT Fast Real-Time PCR System equipment (PE Applied Biosystems, USA) and the ABI PRISM 7900 SDS 2.2.2 Software were used. In all gene expression experiments, cytoplasmic *β*-actin (*Actb*) (VIC/MGB Probe, Primer Limited) was used as the endogenous reference gene (PE Applied Biosystems, USA), All reactions were performed using the TaqMan Gene Expression Master Mix (PE Applied Biosystems, USA) and the TaqMan Gene Expression Assays (FAM) according to the instructions of the equipment and reagent manufacturers. All samples to be compared were run in the same experiment and every reaction was run in quadruplicate. The amount of the target gene was compared to the housekeeper gene by means of the 2^−ΔCT^ method [39]. The following TaqMan Gene Expression Assays (FAM) were used: Ppard (Mm00803184_m1); Fmo2 (Mm0049019_m1); Sult3a1 (Mm00491057_m1); Lepr (Mm0040181_m1); Wfs1 (Mm01220326_m1).

### 2.6. Statistics

Data are presented as means ± SEM and were compared by two-way analysis of variance (ANOVA, treatment and genotype as the independent factors) followed by Tukey's post hoc test. A *P* value of <0.05 was considered statistically significant. Statistical analysis was performed using STATISTICA version 9 (StatSoft Ltd., Bedford, UK) and GraphPad Prism version 5 software (GraphPad Software Inc., San Diego, CA, USA).

## 3. Results

### 3.1. Description of* Wfs1* KO mice

**Figure 1:**
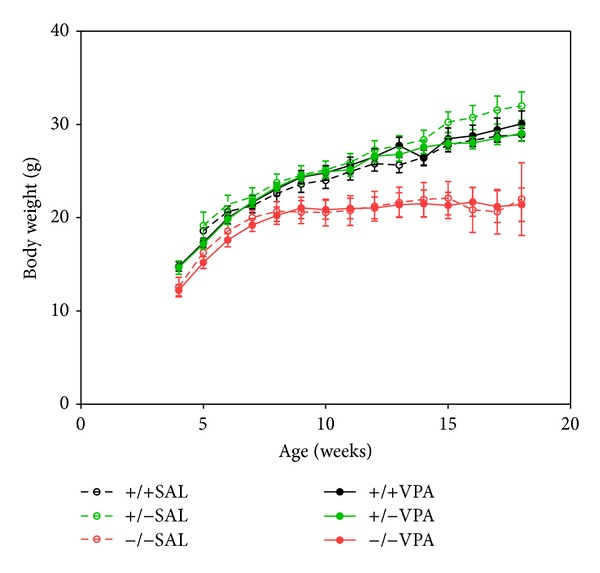
Body weight of male WT and* Wfs1* mutant mice. At the age of 16 months,* Wfs1* KO (red circles) had lower body weight than WT (black circles) or heterozygous HZ (green circles) mice (*F*(2,35) = 7.97; *P* = 0.0014). Growth rate of* Wfs1* KO mice was inhibited since 8th to 9th week of age, while the body weight of WT and HZ continued to increase (*F*(24, 420) = 9.65; *P* < 0.000001). Chronic administration of VPA for 3 months (300 mg/kg/day, i.p. solid symbols) had no effect on growth rate regardless of genotype (*F*(1, 35) = 1.43; *P* = 0.2393). Data is presented as mean ± SEM (*n* = 8).

To determine the effect of VPA treatment on growth, the weight of WT,* Wfs1* HZ, and* Wfs1* KO mice was recorded weekly for 14 weeks. At the age of 16 weeks, homozygous* Wfs1* KO mice had a remarkably lower mean body weight than wild type (WT) or heterozygous (HZ) mice (*F*(2,35) = 7.97; *P* = 0.0014) (Figure 1). There were noticeably different growth rates between the genotypes starting from 8th to 9th week of age, when the growth of* Wfs1* KO was retarded, while the body weight of WT and HZ continued to increase (*F*(24, 420) = 9.65; *P* < 0.000001). Chronic administration of VPA for 3 months had no effect on growth regardless of genotype, confirmed by Tukey**'**s HSD (*F*(1, 35) = 1.43; *P* = 0.2393).

### 3.2. Glucose Tolerance Test after Chronic VPA Treatment

**Figure 2:**
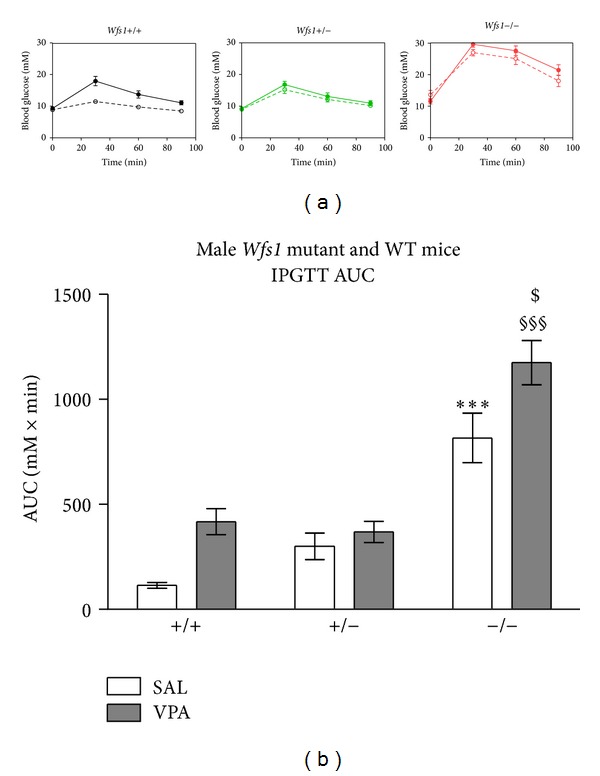
Glucose tolerance test in male WT and* Wfs1* mutant mice after 3-month VPA treatment (300 mg/kg/day). (a) Time course of blood glucose levels following glucose challenge (2 g/kg, i.p.). Blood glucose was measured from tail vein immediately before and 30, 60, and 90 minutes following glucose administration. VPA treatment (solid circles) had no effect on glucose tolerance in* Wfs1* KO or* Wfs1* HZ mice when compared to respective vehicle group (0.9% saline, 10 mL/kg, i.p., open circles). (b) Area under the curve of glucose time curves. Two-way ANOVA followed by Tukey's HSD test (***P < 0.001 versus (+/+SAL); ^§§§^ *P* < 0.001 versus (+/+VPA); ^$^ *P* < 0.05 versus (−/−SAL)). Data is presented as mean ± SEM (*n* = 8).

Basal blood glucose levels of saline treated mice were slightly but significantly elevated in the KO group as compared to the WT or HZ group (*F*(2,21) = 10.03; *P* = 0.00088). Administration of glucose (2 g/kg i.p.) induced a rise in blood glucose levels with a peak at 30 min following glucose administration in all genotypes (Figure 2(a)); this increase was the highest in* Wfs1* KO mice (*F*(2,21) = 75.71; *P* = 0.000001). Tukey's HSD test confirmed peak blood glucose levels of the KO group being significantly higher compared to the WT or HZ group (*P* = 0.00014); also blood glucose levels in the homozygous group were higher than in WT (*P* = 0.026).

Chronic administration of VPA had no effect on the basal blood glucose levels regardless of genotype (*F*(1,43) = 0.52; *P* = 0.475) but resulted in an increase of peak blood glucose concentration at 30 min in WT but not in* Wfs1* HZ or KO mice (*F*(1,43) = 17.31; *P* = 0.00015). Tukey's HSD test confirmed peak blood glucose levels of VPA treated WT mice being significantly higher than in saline treated WT mice (*P* = 0.0014). VPA had no effect on peak blood glucose concentration in* Wfs1* KO or HZ mice. However, there was no statistically significant effect of VPA treatment-genotype interaction (*F*(2,43) = 2.867; *P* = 0.0677).

*Wfs1* KO mice had largest area under the curve (AUC) of IPGTT test (*F*(2,43) = 52.81; *P* = 0.000001), Tukey's HSD test confirmed* Wfs1* KO mice having greater AUC than WT (*P* = 0.00014) or HZ mice (*P* = 0.00068). There was no difference of AUC values between WT and* Wfs1* HZ group. Chronic administration of VPA resulted in an increase of AUC (*F*(1,43) = 14.16; *P* = 0.0005); Tukey's HSD test confirmed VPA treated* Wfs1* KO mice having greater AUC than saline treated* Wfs1* KO mice (*P* = 0.025). Chronic treatment with VPA had no effect on AUC values in WT (*P* = 0.099) and* Wfs1* HZ mice (*P* = 0.99).

### 3.3. Hepatic Gene Transcription Profile

Total RNA was extracted from the liver of male mice and analysed using the Affymetrix GeneChip Mouse Gene 1.0 ST Array. Quality of microarray hybridization and distribution of raw signal intensity across microarray chips was uniform across 48 samples (data not shown).

We did not see a decrease in expression of* Wfs1* gene in* Wfs1* KO mice using gene chip array. Affymetrix GeneChip Mouse Gene 1.0 ST Array has a probe for every exon of the gene. Our* Wfs1* mutant mouse was created by invalidating just two exons (7 and 8) of the* Wfs1* gene; the remaining six exons of this gene are intact in* Wfs1* KO mice. Thus, we did not detect decreased expression levels of* Wfs1* gene in* Wfs1* KO mice using these arrays. However, exon specific analysis revealed lower expression of exons 7 and 8 of* Wfs1* gene in* Wfs1* KO mice (data not shown).

We identified large number of genes that are differentially expressed depending on* Wfs1 *genotype. There were 23 upregulated and 19 downregulated genes in* Wfs1* KO mice as compared to WT mice (Table 1). Ten genes were regulated by VPA treatment (Table 2) and further 9 genes showed an interaction between genotype and VPA treatment (Table 3).

### 3.4. Confirmation of Selected Hits by qRT-PCR

**Figure 3:**
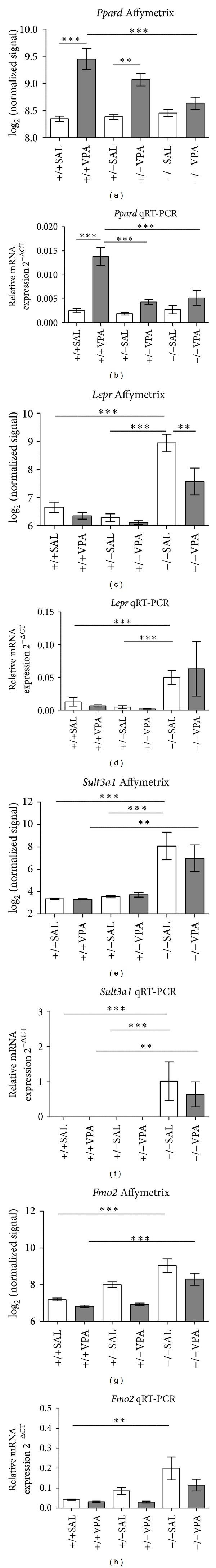
Comparison of results from Affymetrix GeneChip Mouse Gene 1.0 ST Array and qRT-PCR analysis. qRT-PCR mRNA expression is represented as the mean of quadruplicate per sample against the endogenous reference gene* ACTB*. (a) and (b) Ppard. Upregulation of* Ppard* by valproic acid (VPA) is abolished by* Wfs1* invalidation. (c) and (d) Lepr.* Lepr* is upregulated in* Wfs1* KO mice. (e) and (f) Sult3a1.* Sult3a1* expression is increased in* Wfs1* KO mice while downregulated by VPA. (g) and (h) Fmo2.* Fmo2* expression level is increased in* Wfs1* KO mice. All data are presented as means ± SEM (*n* = 8) and were compared by two-way analysis of variance (ANOVA) followed by Tukey's post hoc test (***P < 0.001, **P < 0.01).

The change in expression levels of selected genes (*Sult3a1*,* Fmo2*,* Lepr,* and* Ppard*) was verified with qRT-PCR technique (Figure 3). qRT-PCR data showed similar expression levels as Affymetrix gene chip analysis (Figure 3).

The expression level of* Ppard* was elevated by VPA treatment (*F*(1,42) = 52.5; *P* < 0.000001), and the effect was dependent on genotype (*F*(2,42) = 8.66; *P* = 0.0007) as revealed by Affymetrix GeneChip data (Figure 3(a)). The induction of* Ppard* expression by VPA was strongest in WT mice and lowest in* Wfs1* KO mice. Similar results were obtained also by qRT-PCR analysis for effect of VPA treatment (*F*(1,42) = 36.34; *P* < 0.0000001) and treatment-genotype interaction (*F*(2,42) = 10.8; *P* = 0.0002, Figure 3(b)).

Expression of* Lepr* was highly elevated in liver of* Wfs1* KO mice in comparison to WT or* Wfs1* HZ mice (*F*(2,42) = 38.8; *P* < 0.000001) as revealed by Affymetrix GeneChip data (Figure 3(c)). The effect of genotype on* Lepr* expression pattern was confirmed with qRT-PCR method (*F*(2,38) = 12.95; *P* = 0.00005, Figure 3(d)). Two-way ANOVA revealed also inhibitory effect of VPA on the expression of* Lepr* according to Affymetrix GeneChip data (*F*(1,42) = 8.5; *P* = 0.0057); however, such effect of VPA treatment was not confirmed by qRT-PCR analysis, possibly due to a large variation in* Wfs1* KO VPA group in qRT-PCR analysis (Figure 3(d)).

Expression level of* Sult3a1* in liver of male* Wfs1* KO mice was much higher than in male* Wfs1* HZ or male WT mice (*F*(2,42) = 22.5; *P* < 0.00001) as revealed by Affymetrix gene chip, such finding was confirmed by qRT-PCR analysis (*F*(2,37) = 6.34; *P* = 0.004). In fact, the expression level of* Sult3a1* in WT mice was below detection limit by qRT-PCR method. VPA treatment had no effect on expression level of* Sult3a1 *(Figures 3(e) and 3(f)).

The expression level of* Fmo2* was dependent on genotype and VPA treatment as revealed by Affymetrix gene chip analysis (Figure 3(g)); its expression was higher in* Wfs1* KO as compared to WT mice (*F*(2,42) = 30.4; *P* < 0.000001). The expression of* Fmo2* was inhibited by VPA treatment in all genotypes (*F*(1,42) = 16.5; *P* = 0.0002). Similar results were obtained also by qRT-PCR analysis for genotype (*F*(2,42) = 10.9; *P* = 0.001) and VPA treatment (*F*(1,42) = 5.04; *P* = 0.03, Figure 3(h)).

### 3.5. Regulation of* Wfs1* Expression by VPA

**Figure 4:**
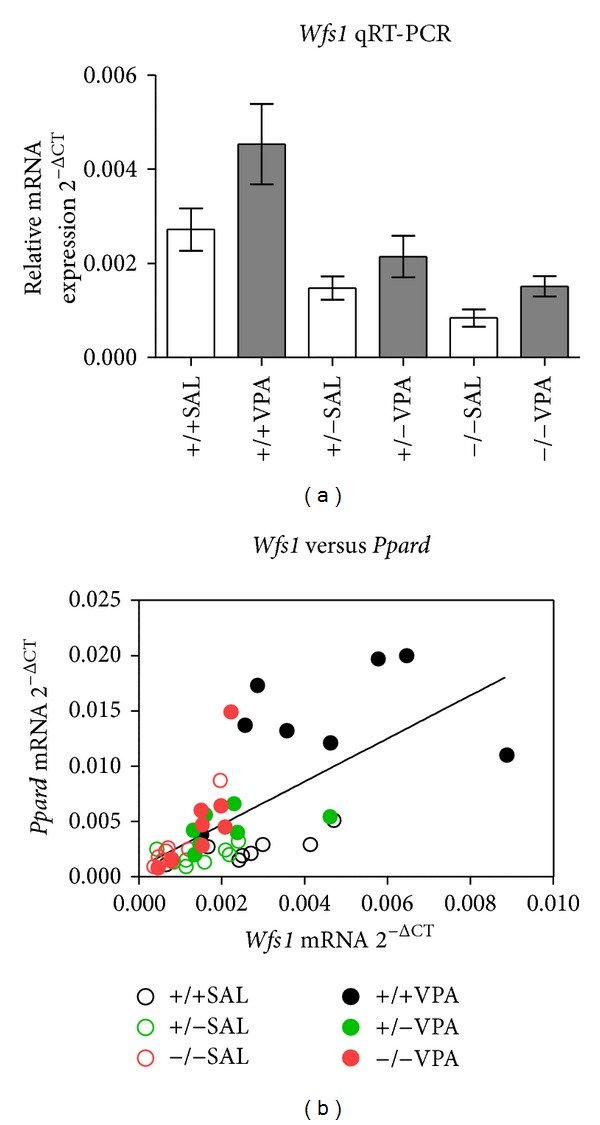
qRT-PCR analysis of* Wfs1* expression in hepatic tissue of WT and* Wfs1* mutant mice. qRT-PCR mRNA expression is represented as the mean of quadruplicate per sample against the endogenous reference gene Actb. (a) Chronic treatment with valproic acid (VPA) induces upregulation of* Wfs1* expression (*F*(1,41) = 7.72; *P* = 0.0082). Expression level of* Wfs1* follows a gene-dose relationship with* Wfs1* genotype (*F*(2,41) = 15.38; *P* < 0.0001). All data are presented as means ± SEM (*n* = 8) and were compared by two-way analysis of variance (ANOVA). (b) Relationship between the expression levels of* Wfs1* and* Ppard*, linear regression across all data points *R* ^2^ = 0.433 (*P* < 0.0001), and expression levels of* Ppard* and* Wfs1* were measured using qRT-PCR, each point corresponds to one animal.

qRT-PCR analysis revealed that the expression level of* Wfs1* was elevated by VPA treatment (*F*(1,41) = 7.72; *P* = 0.0082); however, there was no interaction of treatment with* Wfs1* genotype (Figure 4(a)). As expected, the expression level of* Wfs1* was dependent on genotype (*F*(2,41) = 15.38; *P* < 0.0001); expression level of* Wfs1* in heterozygous mutant mice was reduced to 54% in comparison with wild type mice (Figure 4(a)). The expression level of* Wfs1* was compared to expression level of* Ppard* for possible interaction (expression of both genes was measured by qRT-PCR); regression coefficient of linear regression across all samples was *R* ^2^ = 0.433 (*P* < 0.0001, Figure 4(b)).

## 4. Discussion

The growth of* Wfs1* KO mice was retarded compared to WT or* Wfs1* HZ animals, confirming the results of our previous study [35]. Chronic treatment with VPA had no effect on the growth rate (Figure 1). Interestingly, the growth of* Wfs1* KO mice seems to be similar to that of WT mice until 8 weeks of age, but thereafter the growth of* Wfs1* KO mice slows down, while WT and* Wfs1* HZ mice continue to grow. The mechanism of such age dependency is not known but could be related to the sexual development of mice. We have shown that acute administration of VPA improves glucose tolerance of* Wfs1* KO and HZ mice [36], thus we wanted to evaluate the effect of chronic VPA administration in these mice. Interestingly, chronic treatment with VPA had no effect on basal blood glucose levels regardless of genotype (Figure 2). However, chronic VPA treatment resulted in increased peak glucose level during glucose tolerance test in WT mice and VPA treated* Wfs1* KO mice showed an increased area under the curve during glucose tolerance test. Therefore, chronic VPA seems to impair glucose tolerance of* Wfs1* KO mice, contrary to its acute effect.

We identified a number of genes that are differentially expressed depending on* Wfs1* genotype; a few of them are regulated by VPA treatment. Animals were sacrificed 48 hours after last administration of valproic acid and it is possible that drug treatment effects are normalized during that time. Therefore, the genes showing persistent alteration might be the most relevant ones. We identified 23 upregulated and 19 downregulated genes in* Wfs1* KO mice as compared to WT mice (Table 1). Ten genes were altered by VPA treatment (Table 2) and further 9 genes showed an interaction with genotype and treatment (Table 3). The expression levels of four genes were evaluated by qRT-PCR, and the two methods gave qualitatively similar results.

Many of the genes which were dependent on the* Wfs1* genotype were functionally involved in oxidative processes, including cytochromes, proteins that participate in electron transport (*Cyp2b13*,* Cyp2a22*,* Cyp17a1*,* Cyp2c38*,* Cyp4a14, Cyp8b1*,* Cyp2u1*,* Cyp4a12b*, and* Cyp7b1*), but also genes for monooxygenases (*Fmo3* and* Fmo2*) and organic anion transporters (BC014805, AB056442,* Abcb1a*, and* Slco1a4*). Some of the genes that were upregulated in* Wfs1* KO mice are involved in lipid metabolism (*Hao2*,* Lepr*,* Pnpla3*,* Acot3*, and* Ppargc1a*). Also, some of the genes with decreased expression in* Wfs1* KO mice are involved in fatty acid metabolism (*Rarres1*,* Fitm1*,* Hsd3b5*, and* Elovl3*). Interestingly, earlier reports found* Elovl3* to be upregulated in the liver in response to subchronic [40] and single administration of VPA [41].

The main aim of this study was to identify genes for which the regulation by chronic VPA treatment is dependent on the* Wfs1* genotype. The three genes with the largest change in expression were peroxisome proliferator activator receptor delta (*Ppard*), interleukin-3 regulated nuclear factor (*Nfil3*), and nuclear receptor subfamily 1, group D, member 2 (*Nr1d2*); all of them are also linked with circadian rhythms [42].

In WT mice, VPA treatment caused an approximately 2-fold increase in the expression of* Ppard* compared to vehicle treatment. Such VPA induced upregulation of* Ppard* was not detected in the liver of* Wfs1* KO mice. These results were verified by qRT-PCR analysis (Figures 3(a) and 3(b)). Moreover, similar effect of VPA on* Ppard* expression was observed earlier using* in vitro* bioassays in CHO and F9 cell lines, where VPA activates* Ppard* gene expression [43, 44]. Thus, this finding is in agreement with earlier studies. Based on PPAR reporter assays, VPA is classified as a “triple ppar-alpha, -beta/delta, -gamma agonist” [45].* Ppard* regulates the expression of its target gene* Pdk1* [46]; it is noteworthy that the expression of this kinase was found to be upregulated in response to VPA treatment in the liver slices [45].

PPARs are lipid-activated nuclear receptors with several physiological functions, including control of fatty acid metabolism in different tissues [47]. There are three different subtypes of PPAR: PPAR*α*, PPAR*β*/*δ* (*PPARD*), and PPAR*γ*, each having different expression and biological activities [48]. PPAR*α* is mainly expressed in tissues with intensive *β*-oxidation such as liver, kidneys, heart, skeletal muscles, and intestine. It has been apparent from the experimental and clinical trials that PPAR*α* is important for fatty acid oxidation in the liver and heart [48]. PPAR*γ* participates in the proliferation and differentiation of adipocytes. It is mainly expressed in the fat tissue, colon, endothelial cells, and in the smooth muscle cells of blood vessels [48–50].* PPARD* is widely expressed, but its physiological roles are not as well understood as the ones of the other subtypes. PPARD participates in the skin healing process and is also important in controlling fatty acids oxidation in several tissues, for example, muscle and fat tissue [47, 51]. Recently, an intriguing role of PPARD in the regulation of hepatic lipogenic pathway and fat use by muscle was identified. Liver-specific PPARD activation increases fatty acid uptake in the muscle via regulation of circulating fatty acids [52]. In addition, PPARD activation intensifies glycolysis and the work of pentose phosphate shunt and promotes fatty acid synthesis [48, 53].

Activation of* PPARD* has beneficial effect on body weight and is proposed as treatment of type 2 diabetes [53]. Interestingly, mutations in the* PPARD* gene are associated with BD in the American population [54]; thus there might be also a direct deficit of* PPARD* in patients with BD leading to the development of metabolic syndrome. Activation of PPARD in respective patients needs to be measured to test such a hypothesis.

PPARD agonists are suggested as potential drugs in the case of overweight and problems associated with that [48, 55]. Moreover, Ppard interaction with hepatic AMPK (phospho-AMP-activated protein kinase), PGC-1*α* (PPAR*α*-PPAR*γ* coactivator), and lipin-1 refers to them as therapeutic targets in the prevention of dyslipidemia [51]. It was recently shown that Ppard agonist GW501516 prevents high fat diet associated hyperglyceridemia [56]. GW501516 also restores hepatic AMPK level, which is decreased with the over-consumption of fat, and enhances lipin-1-PGC-1*α* dependent pathway rising hepatic fatty acids oxidation [56]. Remarkably,* Pparγ*c1a is one of the genes whose expression was significantly higher in* Wfs1* KO mice compared to WT mice (Table 1).

It is unknown whether* Wfs1* is required for VPA mediated induction of* Ppard* expression or lack of VPA effect in* Wfs1* KO mice is caused by some secondary changes in these mice. Chronic VPA treatment results in an increase of* Wfs1* expression (Figure 4(a)). There seems to be a correlation between the expression levels of* Wfs1* and* Ppard* (Figure 4(b)), further suggesting a regulatory link between these two genes. However, molecular studies linking* Wfs1* function with the regulation of* Ppard* are needed to definitely answer this question. Also, it would be most interesting to compare the regulation of PPARD in patients with and without VPA treatment-induced weight gain. Based on our results, we would speculate that PPARD is activated in patients without drug induced weight gain, while its activity is lower in obese patients receiving VPA.

It is hard to predict whether PPARD ligands will eventually be developed into FDA-approved drugs. Results of this study suggest that these drugs must be evaluated for possible interaction with valproic acid before use in patients.

## 5. Conclusions

GeneChip analysis showed that invalidation of the* Wfs1* gene induces changes in liver transcriptome with impact on genes involved in lipid and fatty acid metabolism. Expression of* Ppard* in the liver is upregulated in response to chronic treatment with valproic acid, such upregulation is absent in* Wfs1* KO mice. Importance of Ppard in the regulation of metabolic processes is well recognized; thus the role of such Wfs1-VPA interaction on the regulation of Ppard needs further investigation.

## Figures and Tables

**Table 1:** List of genes whose expression in mouse liver is regulated by *Wfs1* genotype as measured by Affymetrix GeneChip Mouse Gene 1.0 ST Array.

Probeset ID	*P* value	*Q*-value	Fold change	Gene	Gene description
**Upregulated in *Wfs1* KO mice**					
10362464	6.32*E* − 05	0.028767	131.3	Sult3a1	Sulfotransferase family 3A, member 1
10359593	4.94*E* − 06	0.008869	112.2	Fmo3	Flavin containing monooxygenase 3
10551209	3.81*E* − 04	0.055722	37.6	Cyp2b13	Cytochrome P450, family 2, subfamily b, polypeptide 13
10465726	8.82*E* − 06	0.011336	14.0	BC014805	cDNA sequence BC014805
10561162	7.93*E* − 05	0.031687	10.3	Cyp2a22	Cytochrome P450, family 2, subfamily a, polypeptide 22
10465734	3.50*E* − 06	0.008869	9.8	AB056442	cDNA sequence AB056442
10500570	1.07*E* − 04	0.035569	6.3	Hao2	Hydroxyacid oxidase 2
10506301	1.46*E* − 05	0.013481	5.2	Lepr	Leptin receptor
10425822	7.17*E* − 04	0.080582	5.0	Pnpla3	Patatin-like phospholipase domain containing 3
10520622	5.54*E* − 15	1.79*E* − 10	4.8	Abhd1	Abhydrolase domain containing 1
10468239	4.10*E* − 04	0.05785	4.4	Cyp17a1	Cytochrome P450, family 17, subfamily a, polypeptide 1
10359582	9.44*E* − 07	0.004726	4.4	Fmo2	Flavin containing monooxygenase 2
10397158	1.19*E* − 04	0.037527	3.5	Acot3	acyl-CoA thioesterase 3
10574027	7.39*E* − 04	0.081551	3.5	Mt1	Metallothionein 1
10533401	7.42*E* − 04	0.081551	2.9	Cux2	Cut-like homeobox 2
10350733	3.59*E* − 04	0.053424	2.8	Rgs16	Regulator of G-protein signaling 16
10465740	2.81*E* − 04	0.049115	2.4	Gm6192	Predicted gene 6192
10362472	2.97*E* − 04	0.050271	2.2	Rsph4a	Radial spoke head 4 homolog A (Chlamydomonas)
10467372	6.13*E* − 05	0.028301	2.1	Cyp2c38	Cytochrome P450, family 2, subfamily c, polypeptide 38
10519527	1.29*E* − 04	0.038113	2.1	Abcb1a	ATP-binding cassette, sub-family B (MDR/TAP), member 1A
10529977	3.97*E* − 06	0.008869	2.1	Ppargc1a	Peroxisome proliferative activated receptor, gamma, coactivator 1 alpha
10548996	4.56*E* − 04	0.062377	2.0	Slco1a4	Solute carrier organic anion transporter family, member 1a4
10515187	6.50*E* − 04	0.07526	2.0	Cyp4a14	Cytochrome P450, family 4, subfamily a, polypeptide 14

**Downregulated in *Wfs1* KO mice**					
10571560	1.68*E* − 04	0.03933	2.0	Mtnr1a	Melatonin receptor 1A
10597875	1.21*E* − 05	0.01261	2.0	Cyp8b1	Cytochrome P450, family 8, subfamily b, polypeptide 1
10454015	1.42*E* − 05	0.01348	2.0	Ttc39c	Tetratricopeptide repeat domain 39C
10502214	5.25*E* − 06	0.00892	2.2	Cyp2u1	Cytochrome P450, family 2, subfamily u, polypeptide 1
10498584	9.12*E* − 06	0.01133	2.4	Rarres1	Retinoic acid receptor responder (tazarotene induced) 1
10482528	2.47*E* − 05	0.01781	2.5	Neb	Nebulin
10579649	4.58*E* − 05	0.02463	2.6	Cib3	Calcium and integrin binding family member 3
10415279	2.82*E* − 05	0.01939	2.6	Fitm1	Fat storage-inducing transmembrane protein 1
10602372	2.33*E* − 06	0.00740	2.6	Alas2	Aminolevulinic acid synthase 2, erythroid
10548931	1.53*E* − 04	0.03829	2.7	Slc15a5	Solute carrier family 15, member 5
10500545	1.25*E* − 04	0.03811	2.8	Hsd3b5	Hydroxy-delta-5-steroid dehydrogenase, 3 beta- and steroid delta-isomerase 5
10507152	2.10*E* − 04	0.04265	3.0	Cyp4a12b	Cytochrome P450, family 4, subfamily a, polypeptide 12B
10352439	4.13*E* − 06	0.00886	3.1	Susd4	Sushi domain containing 4
10507143	1.99*E* − 04	0.04099	3.1	Cyp4a12a	Cytochrome P450, family 4, subfamily a, polypeptide 12a
10513538	9.25*E* − 05	0.03306	3.1	Mup21	Major urinary protein 21
10545877	4.37*E* − 07	0.00353	3.5	Nat8	N-Acetyltransferase 8 (GCN5-related, putative)
10497381	3.81*E* − 07	0.00353	3.9	Cyp7b1	Cytochrome P450, family 7, subfamily b, polypeptide 1
10545874	5.38*E* − 05	0.02674	4.6	Cml5	Camello-like 5
10463551	1.06*E* − 07	0.00171	6.4	Elovl3	Elongation of very long chain fatty acids (FEN1/Elo2, SUR4/Elo3, yeast)-like 3

**Table 2:** List of genes whose expression in mouse liver is regulated by chronic VPA treatment as measured by Affymetrix GeneChip Mouse Gene 1.0 ST Array.

Probeset ID	*P* value	*Q*-value	Fold change	Gene symbol	Gene description
10454353	1.81*E* − 04	0.66000	1.59	S100a10	S100 calcium binding protein A10 (calpactin)
10425421	2.87*E* − 04	0.738978	1.46	Pcna	Proliferating cell nuclear antigen
10493995	1.60*E* − 04	0.660001	1.46	Fam83f	Family with sequence similarity 83, member F
10518069	9.94*E* − 04	0.754625	1.46	Efhd2	EF hand domain containing 2
10383545	3.52*E* − 04	0.754625	1.45	Pcna	Proliferating cell nuclear antigen
10487930	6.38*E* − 04	0.754625	1.36	Tbc1d19	TBC1 domain family, member 19
10461391	4.38*E* − 05	0.569567	1.35	Gpr39 // Lypd1	G protein-coupled receptor 39 // Ly6/Plaur domain containing 1
10521927	5.10*E* − 05	0.569567	1.35	E130311K13Rik	RIKEN cDNA E130311K13 gene
10349401	4.74*E* − 04	0.754625	1.30	Mocos	Molybdenum cofactor sulfurase
10498477	9.13*E* − 04	0.754625	1.26	Foxk2	Forkhead box K2

**Table 3:** List of genes whose expression in mouse liver by chronic VPA treatment is dependent on *Wfs1* genotype as measured by Affymetrix GeneChip Mouse Gene 1.0 ST Array.

Probeset ID	*P* value	*Q*-value	Fold changes	Gene symbol	Gene description
10443332	1.1*E* − 04	0.99999	2.26	Ppard	Peroxisome proliferator activator receptor delta
10409278	2.1*E* − 03	0.99999	2.17	Nfil3	Nuclear factor, interleukin 3, regulated
10417734	5.6*E* − 04	0.99999	2.03	Nr1d2	Nuclear receptor subfamily 1, group D, member 2
10514520	1.9*E* − 03	0.99999	1.58	Cyp2j9	Cytochrome P450, family 2, subfamily j, polypeptide 9
10535759	9.2*E* − 04	0.99999	1.32	Lnx2	Ligand of numb-protein X 2
10489694	1.9*E* − 03	0.99999	1.26	Zfp334	Zinc finger protein 334
10459353	1.3*E* − 03	0.99999	1.25	Fam38b	Family with sequence similarity 38, member B
10351414	1.7*E* − 03	0.99999	1.20	Aldh9a1	Aldehyde dehydrogenase 9, subfamily A1
10473690	2.1*E* − 03	0.99999	1.18	Fnbp4	Formin binding protein 4
